# PIST (GOPC) modulates the oncogenic voltage-gated potassium channel K_V_10.1

**DOI:** 10.3389/fphys.2013.00201

**Published:** 2013-08-14

**Authors:** Solveig Herrmann, Milena Ninkovic, Tobias Kohl, Luis A. Pardo

**Affiliations:** ^1^AG Oncophysiology, Max-Planck Institute of Experimental MedicineGöttingen, Germany; ^2^Department of Molecular Biology of Neuronal Signals, Max-Planck Institute of Experimental MedicineGöttingen, Germany

**Keywords:** potassium channels, K_V_10.1, KCNH1, PIST, FIG, GOPC, trafficking

## Abstract

Although crucial for their correct function, the mechanisms controlling surface expression of ion channels are poorly understood. In the case of the voltage-gated potassium channel K_V_10.1, this is determinant not only for its physiological function in brain, but also for its pathophysiology in tumors and possible use as a therapeutic target. The Golgi resident protein PIST binds several membrane proteins, thereby modulating their expression. Here we describe a PDZ domain-mediated interaction of K_V_10.1 and PIST, which enhances surface levels of K_V_10.1. The functional, but not the physical interaction of both proteins is dependent on the coiled-coil and PDZ domains of PIST; insertion of eight amino acids in the coiled-coil domain to render the neural form of PIST (nPIST) and the corresponding short isoform in an as-of-yet unknown form abolishes the effect. In addition, two new isoforms of PIST (sPIST and nsPIST) lacking nearly the complete PDZ domain were cloned and shown to be ubiquitously expressed. PIST and K_V_10.1 co-precipitate from native and expression systems. nPIST also showed interaction, but did not alter the functional expression of the channel. We could not document physical interaction between K_V_10.1 and sPIST, but it reduced K_V_10.1 functional expression in a dominant-negative manner. nsPIST showed weak physical interaction and no functional effect on K_V_10.1. We propose these isoforms to work as modulators of PIST function via regulating the binding on interaction partners.

## Introduction

The voltage gated potassium channel K_V_10.1 is the founding member of the family of *ether à go-go channels* (Warmke and Ganetzki, 1994). The function of this ion channel in the central nervous system, where it is normally exclusively expressed, still remains elusive (Occhiodoro et al., 1998; Pardo et al., 1999). Ectopically expressed K_V_10.1 induces a transformed phenotype, and the channel is expressed in 70% extra-cranial tumors (Hemmerlein et al., 2006; Mello De Queiroz et al., 2006; Ding et al., 2007a,b, 2008; Ousingsawat et al., 2007; Wadhwa et al., 2009; Agarwal et al., 2010; Asher et al., 2010). Although there is evidence pointing to an additional non-canonical, permeation-independent role of the channel in cancer initiation and growth (Downie et al., 2008; Chen et al., 2011), inhibition of channel function exclusively at the plasma membrane by a monoclonal antibody (Gomez-Varela et al., 2007) reduces tumor progression *in vivo*, indicating that the membrane population of the channel is very important for its oncogenic properties. However, the mechanisms regulating the expression of K_V_10.1 at the surface are still unclear, although it is known that the membrane residence time of the channel is relatively short, because it is internalized at a rate of 2% per minute (Kohl et al., 2011), and that both rabaptin 5 and cortactin are important to determine the abundance of K_V_10.1 at the plasma membrane at a given time(Herrmann et al., 2012; Ninkovic et al., 2012).

PIST (PDZ domain protein interacting specifically with TC10), also called GOPC (Golgi-associated PDZ and coiled-coil motif-containing protein), CAL (CFTR-associated ligand) or FIG (fused in glioblastoma) was first described in 2001 as a coiled-coil and PDZ domain-containing protein that specifically interacts with the GTPase TC10 (Neudauer et al., 2001). Depending on one of its coiled-coil regions, PIST mainly resides in the Golgi apparatus, but can also co-localize with its binding partners at the plasma membrane (Charest et al., 2001; Yao et al., 2001; Wente et al., 2005b). Many studies have addressed the possible interaction partners of PIST, in order to unravel the function of this protein. Proteins known to be located at the *trans*-Golgi network (TGN) like Syntaxin 6 or Golgin160 are able to bind the coiled-coil region of PIST, pointing to an involvement of this protein in processes like TGN sorting and vesicle trafficking (Charest et al., 2001; Hicks and Machamer, 2005). A crystal structure of this domain of PIST is available (Shin et al., 2013). The ability of PIST to bind activated small GTPases like TC10 and Rab6a may serve as a mechanism controlling PIST function in response to signaling pathways, as shown for cystic fibrosis transmembrane conductance regulator (CFTR) (Neudauer et al., 2001; Cheng et al., 2005; Bergbrede et al., 2009). Most other interaction partners of PIST are reported to bind to its PDZ domain, among them CFTR itself (Cheng et al., 2002), the G-protein-coupled receptors *frizzled* 5 and 8 (Yao et al., 2001), the chloride channel CIC-3B (Gentzsch et al., 2003), the β 1-adrenergic receptor (He et al., 2004), the somatostatin receptor subtype 5 [SSTR5 (Wente et al., 2005b)], a metabotropic glutamate receptor [mGluR1a, (Zhang et al., 2008)] and Cadherin 23 (Xu et al., 2010). Several studies discuss that overexpression of PIST might lead to different surface expression patterns of some of these membrane proteins by holding them back in the Golgi (He et al., 2004; Wente et al., 2005b; Xu et al., 2010). A different and more detailed process is postulated for CFTR, where PIST enhances degradation by facilitating its targeting to lysosomes (Cheng et al., 2005). A fusion between PIST and the proto-oncogene ROS1 has been detected, initially in glioblastoma and subsequently in other cancer types (Charest et al., 2003; Birch et al., 2011; Gu et al., 2011; Suehara et al., 2012).

Relevant functions of PIST in the brain appear to be mediated by an alternatively spliced isoform (nPIST) that contains an eight amino acid insertion in the second coiled-coil region (Yue et al., 2002). This isoform is able to interact with Beclin1 over its coiled-coil domain and therefore was linked to autophagy in neurons of lurcher mice (Yue et al., 2002), although constitutive ion fluxes were able to induce cell death regardless of the nPIST-Beclin1 interaction (Nishiyama et al., 2010). Additionally, glutamate receptors are able to bind to the PDZ domain of nPIST over their extreme C-terminus (Yue et al., 2002; Cuadra et al., 2004). This interaction in concert with Stargazin has been shown to enhance synaptic clustering of AMPA receptors in hippocampal neurons (Cuadra et al., 2004).

In this study, we identified PIST as a new interaction partner of K_V_10.1. We show physical as well as functional interaction between PIST and K_V_10.1. We were also able to identify and clone two new isoforms of PIST, and provide evidence that the four isoforms of PIST differ in terms of binding to and regulation of K_V_10.1.

## Materials and methods

### Yeast two-hybrid

The yeast reporter strain L40 (Vojtek et al., 1993) (*MATa, trp1, leu2, his3, LYS::lexA-HIS3, URA3::lexA-lacZ*) was transformed with pLexN-hEag1 by the lithium acetate method and grown on synthetic medium lacking tryptophan. After additional transformation with plasmid pVP16-3-cDNA [postnatal 8 rat brain cDNA library, (Okamoto and Sudhof, 1997)], double transformants were plated on synthetic medium lacking histidine, leucine, uracil, lysine, tryptophan and in the presence of the competitive inhibitor of the HIS3 protein 3-amino-1,2,4-triazole (3-AT). Positive colonies were picked after 4–6 days and tested for ß-galactosidase activity using plate assay. Plasmids from positive clones were rescued and transformed in *E. coli* strain HB101. *E. coli* cells were plated on leucine-lacking medium. Positive clones were further analyzed by yeast retransformation and DNA sequencing.

### RNA purification, cRNA synthesis and RT-PCR

HEK 293 cells were washed 3 times with ice cold PBS, harvested and directly used for RNA purification using RNAeasy Mini Kit (Qiagen). First strand cDNA was produced using SuperScript (Invitrogen) with oligo-dT. For cloning, PIST was amplified in a standard PCR reaction using Taq DNA polymerase (NEB) and sense and antisense primers (see below). PCR products were analyzed by electropohoresis, fragments excised out of gel and purified using NucleoSpin Extract (Macherey-Nagel) and subcloned in pGEMT-easy (Promega) following manufacturer's instructions.

Mouse first strand cDNA was a generous gift from Dr. R. Ufartes (Ufartes et al., 2013).

To distinguish between isoforms, PCR products were obtained in a standard PCR procedure using 100 ng cDNA mouse first strand cDNA, and a set of 2 or 3 specific primers in the appropriate combinations to distinguish between isoforms (see below) and Biotherme Polymerase (Genecraft).

Primers for human PIST (accession numbers NM_001017408.2—PIST- and NM_020399.3—sPIST-) were:

5′ ATGTCGGCGGGCGGTCCATGC3′ and
5′TTAATAAGATTTTTTATGATACAGAG3′

For mouse PIST (accession numbers NM_001199272.1 and NM_053187.3):

5′GCAGAGGGCGCAACGACTT3′ and
5′ATTCTCATGCGCATCCCTCACTG3′

For selective amplification of the previously described transcript variants, we used different forward primers (nPIST; NM_020399.3: 5′ CAAGGCAAAATTGTCTGTCCAC 3′; PIST, NM_001017408.2: 5′ CAAGGCAAAATTGGAAAGAGAAC 3′), and to distinguish between short and long forms, the following antisense primers were used:

Short 5′ CTCTCTGCTGGGAGAGTATAG 3′
Long 5′ CTCTCCTCTCTGTGTGATAGA 3′

### Cell culture and transfection

HeLa cells were cultured in MEM+GlutaMax (Invitrogen) supplemented with 10% FCS (PAA), HEK293 cells in DMEM/F12+GlutaMax (Invitrogen) supplemented with 10% FCS and for stable cell line HEK293-K_V_10.1-BBS [HEK-BBS, (Kohl et al., 2011)] with Zeocin (Invitrogen) at 5% CO_2_ and 37°C.

Transfection was performed using Lipofectamine 2000 or Lipofectamine (Invitrogen) according to the manufacturer's instructions. K_V_10.x-BBS Venus and CFP-PIST were generated by cloning into pcDNA3 or pECFP-N1 vectors. Empty vectors were used as controls.

### Fractional labeling, quantification and purification of K_V_10.1-BBS

Labeling of whole-cell K_V_10.1-BBS was performed in cell lysates in buffer LP [20 mM Tris-HCl pH 7.4, 150 mM NaCl, 5 mM MgCl_2_, 1% Nonidet P40, protease inhibitors (Roche)] with α-bungarotoxin (BTX)-biotin conjugate (Invitrogen) in a final concentration of 0.2 μg/ml for 30 min on ice. To detect membrane and/or internalized K_V_10.1-BBS, transfected HEK-BBS cells were incubated in media supplemented with α-BTX-biotin conjugate (Invitrogen) in a final concentration of 2.5 μg/ml and kept at room temperature for 10 min (membrane) or at 37°C for one hour (internalized). For internalized K_V_10.1-BBS, cells were washed with ice-cold acid wash buffer (150 mM NaCl, pH 3.0) for 3 min to remove membrane labeling of K_V_10.1-BBS. Twice washing with cold PBS removed residual α-BTX-biotin conjugate. Cells were then harvested and lysed with LP buffer for 20 min on ice. Insoluble fraction was removed by centrifugation at 18,000 × *g* at 4°C, and the supernatant used for ELISA or pull down experiments.

For pull down approaches, labeled K_V_10.1-BBS was bound on streptavidin-coated magnetic beads (T1, Invitrogen) for 2 h at 4°C. Unbound protein was removed by washing 5 times with buffer LP supplemented with protease inhibitors (Roche). Bound protein was eluted at 70°C for 10 min using LDS sample buffer containing reducing agent (Invitrogen) and analyzed by SDS PAGE (Invitrogen) and western blotting.

Quantification of the amount of labeled K_V_10.1-BBS was performed by ELISA. After labeling, total cell lysates (30 and 150 μg protein), were immobilized on streptavidin-coated plates (Pierce) and detected using a C-terminal monoclonal anti-K_V_10.1 antibody [Ab33, 5 μg/ml (Hemmerlein et al., 2006)] and a polyclonal anti-mouse secondary antibody (Pierce, 1:500) coupled to peroxidase. ABTS (Invitrogen) was used as a substrate for development and detected in a Wallac Victor2 reader at 405 nm (reference 490 nm). Experiments were performed in duplicates.

### Pull down experiments

For immunoprecipitation, rat brain lysates (800 μg total protein) were incubated overnight at 4°C with 2–5 μg of antibody [anti-K_V_10.1 33/62 (Hemmerlein et al., 2006); anti-PIST (Millipore); non-specific mouse IgGκ 2b) in buffer A (0,5% Triton X-100, 25 mM TrisHCl pH7.5, 75 mM NaCl, 2.5 mM EDTA and protease inhibitors (Roche)]. Pull down was performed by adding 20 μl of Protein G/A-coated Sepharose beads (Calbiochem) for 2 h at 4°C under rotation. After washing five times with buffer A, bound protein was eluted at 70°C for 10 min using LDS sample buffer containing reducing agent (Invitrogen) and analyzed by SDS PAGE (Invitrogen) and western blotting.

### Electrophysiology

Recordings on *Xenopus* oocytes were performed as described by Stuhmer (1998) using a Turbo TEC-10CD amplifier (NPI electronics). cRNA was synthesized using the mMessage mMachine kit (Ambion) according to the manufacturer's instructions. 0.1–1 ng cRNA per oocyte were microinjected 1–3 days prior to recording. Cells were kept at 18°C in ND 96 solution (96 mM NaCl, 2 mM KCl, 0.2 mM CaCl_2_, 2 mM MgCl_2_, 5 mM Hepes/NaOH, pH 7.5) supplemented with theophylline (0.5 mM) to avoid maturation of the oocytes. For voltage clamp recordings, pipettes had resistances ranging from 0.5 to 1.2 MΩ when filled with 2 M KCl. External solution (NFR) contained 115 mM NaCl, 2.5 mM KCl, 1.8 mM CaCl_2_, 10 mM Hepes/NaOH, pH 7.2. Current was digitized at 5 kHz and filtered at 1 kHz. Currents were elicited by 1 s depolarizations from a holding potential of −100 mV to values ranging from +80 to −100 mV. Current amplitude was determined as mean value in the last 200 ms of the depolarization. To fit current-voltage relationships, we used an equation of the form:

$$I(V)=\Gamma \cdot \frac{\left(V-V_{rev}\right)}{{\left(1+e^{\frac{V_{50}-V}{k}}\right)}^{4}}$$

where Γ is the total conductance, *V*_*rev*_ the reversal potential, *V*_50_ the potential of half activation per subunit and *k* the slope factor.

Data acquisition and analysis was performed with Pulse-PulseFit (HEKA Electronics) and IgorPro (WaveMetrics).

### Microscopy

For confocal microscopy, HeLa cells were grown on glass coverslips and transfected with Venus-tagged K_V_10.1 and CFP-tagged isoforms of PIST. Empty vectors served as control. 24 h after transfection, the Golgi marker BODIPY FL C_5_ Ceramide (Invitrogen) was used according to manufacturer's instructions to visualize Golgi apparatus. Cells were fixed in 4% paraformaldehyde in PBS for 10 min at room temperature, washed thoroughly with HBBS and mounted with ProLong Gold Antifade Reagent (Invitrogen). Image acquisition was performed using a Zeiss LSM 510 Meta confocal microscope with a Zeiss 40x 1.2 Korr water immersion objective. Single channel gray scale images are presented using color tables according to the fluorophore used. Merged images were generated by combination of the channel images into a single RGB file. Only linear contrast enhancement and Gaussian blur were applied off-line to the images; analysis was carried out with Fiji (Schindelin et al., 2012).

## Results

### PIST interacts with the voltage-gated ion channel K_V_10.1

Finding new interaction partners of K_V_10.1is crucial to unravel the role of this ion channel both in the healthy brain and in tumors. To identify candidate interaction partners of K_V_10.1, we performed yeast two-hybrid screens using a rat brain expression library. The C-terminus of K_V_10.1 in fusion to the DNA binding domain of LexA transcription factor was used as a bait to screen a rat brain cDNA library fused to the activation domain of VP16 transcription factor. As previously reported (Ninkovic et al., 2012), out of 2 million screened clones, eight were positive for the HIS3 marker. One of them encoded most of the open reading frame of the murine PIST, including its PDZ domain (Neudauer et al., 2001).

**Figure 1:**
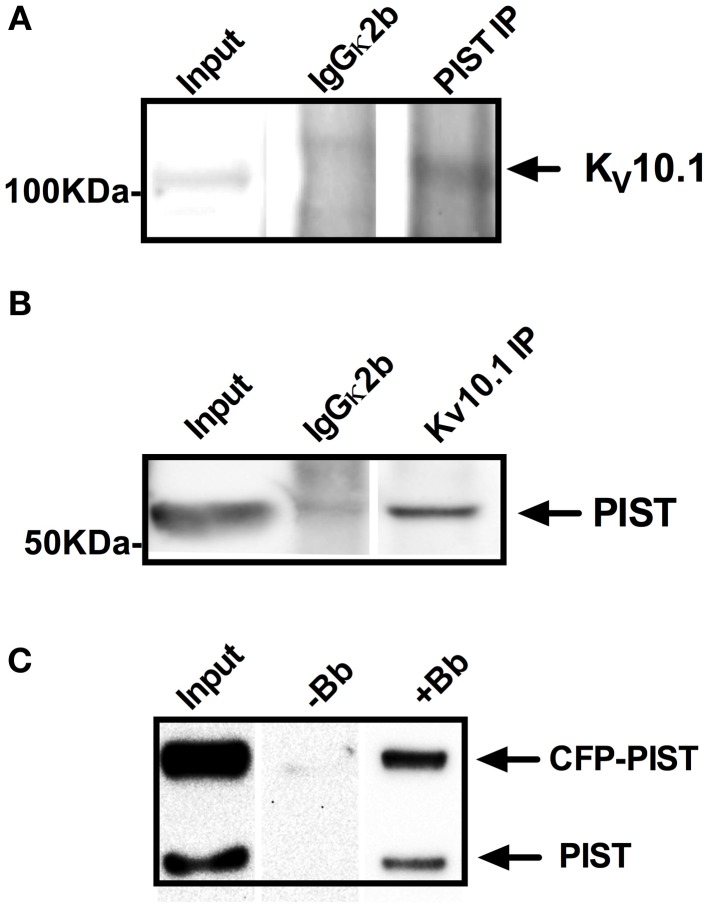
**Co-precipitation of K_V_10.1 and PIST out of (A,B) rat brain lysates and (C) HEK293_K_V_10.1_BBS cells.** Specific antibodies against PIST **(A)** or K_V_10.1 **(B)** were used for precipitation out of rat brain lysates using Protein A/G coupled sepharose beads. Isotype control antibodies served as negative controls. Co-precipitation of Kv10.1 by anti PIST immunoprecipitation **(A)** as well as precipitation of PIST by anti K_V_10.1 immunoprecipitation **(B)** were detected by western blot. **(C)** K_V_10.1_BBS was labeled with an α-bungarotoxin-biotin conjugate after lysis of HEK293_K_V_10.1_BBS cells and pulled down with streptavidin-coated beads. Detection on a western blot with and anti-PIST antibody shows bands compatible with precipitation of both CFP-tagged and endogenous PIST.

We then performed co-immunoprecipitation studies to confirm the interaction. Precipitation of PIST using a specific antibody was able to pull down K_V_10.1 out of rat brain lysates (Figure 1A). In reverse co-IP experiments, PIST co-precipitated K_V_10.1 (Figure 1B). Non-specific isotype antibodies did not precipitate either of the two proteins.

In a second pull-down approach we used HEK293 cells stably expressing a modified K_V_10.1 bearing an α-bungarotoxin (BTX) binding site [K_V_10.1_BBS, (Kohl et al., 2011)]. Specific binding of a BTX-biotin conjugate to its binding site allows precipitation of K_V_10.1_BBS from cell lysates. After transfection with CFP-labeled PIST, pull down of K_V_10.1_BBS with BTX-biotin and immunoblotting using anti-PIST antibodies revealed two bands compatible with endogenous PIST as well as CFP-labeled PIST (Figure 1C, lane +Bb); no bands were detected if BTX-biotin was omitted (Figure 1C, lane −Bb). The fact that both endogenous and CFP-tagged PIST are detected argues in favor of the specificity of PIST detection. As a control, the same experiments using unlabeled K_V_10.1_BBS did not precipitate PIST, also indicating specificity of the co-IP. Taken together, these results strongly indicate that PIST and K_V_10.1 interact physically both *in vitro* and *in vivo*.

### Two new short PIST isoforms lacking the PDZ domain are expressed in all tissues tested

**Figure 2:**
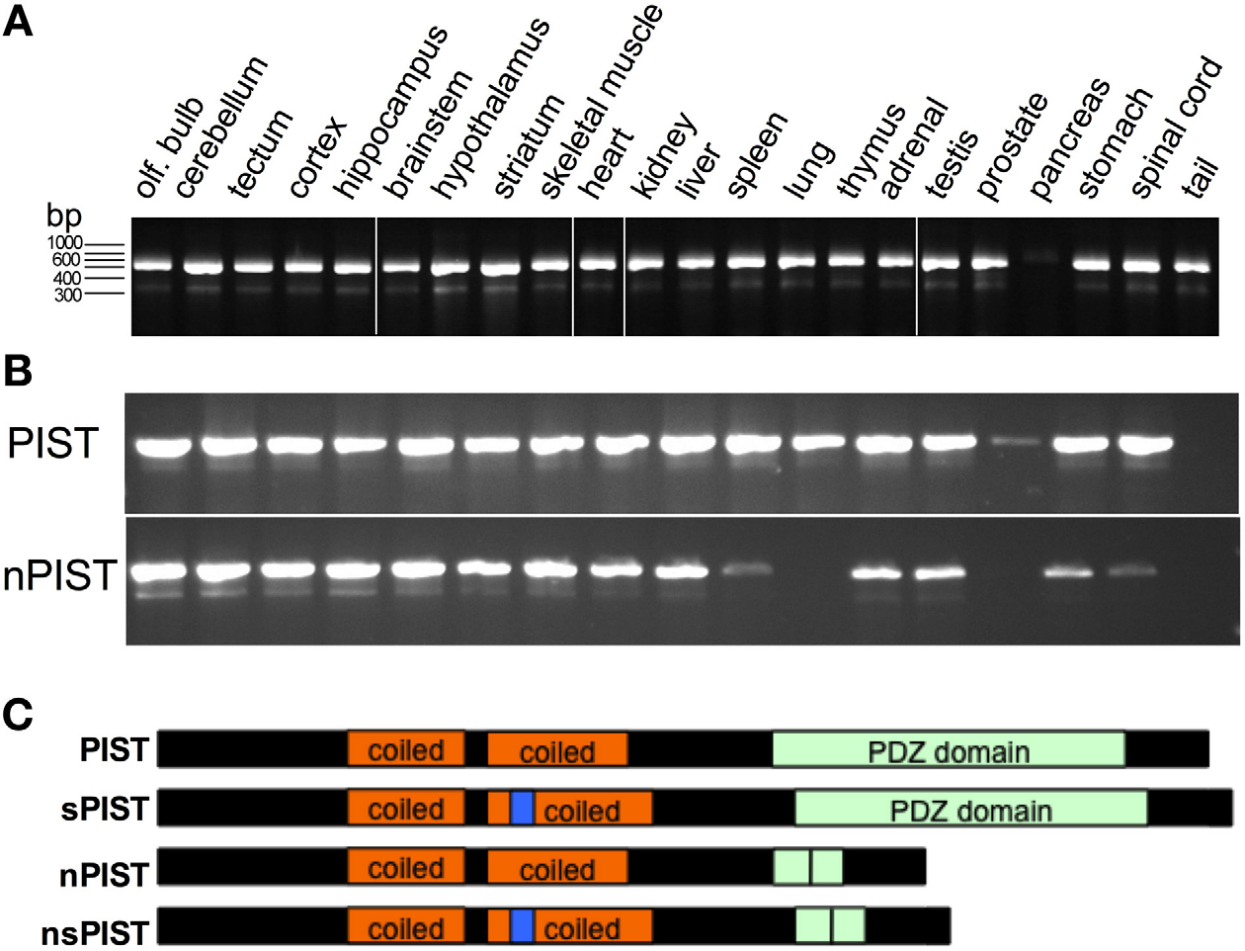
**New PIST isoforms. (A)** PCR on cDNA from different mouse tissues show amplification of short (205 bp) and a long (408 bp) fragments corresponding to sPIST and PIST. **(B)** A PCR designed to differentiate neuronal and non-neuronal forms of PIST detect both short and long bands revealing the widely extended expression of both forms. **(C)** Schematic representation of the domains of all four PIST isoforms. Coiled-coil regions are depicted in orange, the insertion in n-PISTs in the second coiled-coil is represented in blue. The PDZ domain is represented in green, and a vertical line represents the site of deletion of exon 6.

For further analysis we amplified and cloned the human PIST out of cDNA obtained from untransfected HEK293 cells, using 5′ and 3′ flanking primers. Interestingly, during the cloning process we systematically observed two bands, one of them at the expected size of full length PIST and a shorter one. Cloning and subsequent sequencing of the shorter band revealed that it corresponds to a PIST variant lacking the complete exon 6, which is the region coding for the majority of the PDZ domain indicating that this clone corresponds to a new splice variant of PIST. To further proof the existence of the new isoform, we designed primers to generate an amplicon containing the skipped exon, in order to discriminate between the longer and the shorter form of PIST. We were able to amplify fragments with the expected sizes out of cDNA from HEK293 cells (data not shown). To evaluate the tissue distribution of the splice variant we used mouse cRNA from different tissues. The variant (lacking in the mouse exon 5) was detected in all tissues tested except pancreas, where amplification of full-length PIST was also much weaker (Figure 2A). The signals for the short form were consistently weaker than those for full-length PIST.

In 2002, a second isoform of PIST was cloned and termed “neuronal PIST” (nPIST) because it is believed to be the main form in the brain. It differs from PIST by an 8 amino acid insertion in the coiled-coil region (Yue et al., 2002). The sequence we identified here codes for a protein that lacks the main part of the PDZ domain but bears the 8 amino acids of the neuronal form. We therefore asked if there is a fourth isoform, lacking the PDZ as well as the 8 amino acid insertion. PCR with primer pairs distinguishing between nPIST and PIST and producing different fragment length depending on the presence of the alternatively skipped exon was used to answer this question. Amplification of the expected fragments out of cDNA from HEK293 cells as well as from different murine tissues indicated that all four isoforms are expressed in nearly every tissue (Figure 2B). Weak or absent expression of nPIST in liver and pancreas correlated with no detection of the short form (nsPIST) of it, indicating that expression of the shorter form depends on the expression of the corresponding longer one (Figure 2B). The cartoon in Figure 2C depicts the domain structure of the four PIST isoforms. Taken together, our data suggests that two new short forms of PIST (from here on termed sPIST and nsPIST) exist and are expressed in nearly every tissue. According to our semi-quantitative approach, both seem to be less abundant than their corresponding longer form. sPIST and nsPIST sequences have been deposited under accession numbers KF420122 and KF420123, respectively.

### Short and long isoforms of PIST have different capabilities for binding ToK_V_10.1

**Figure 3:**
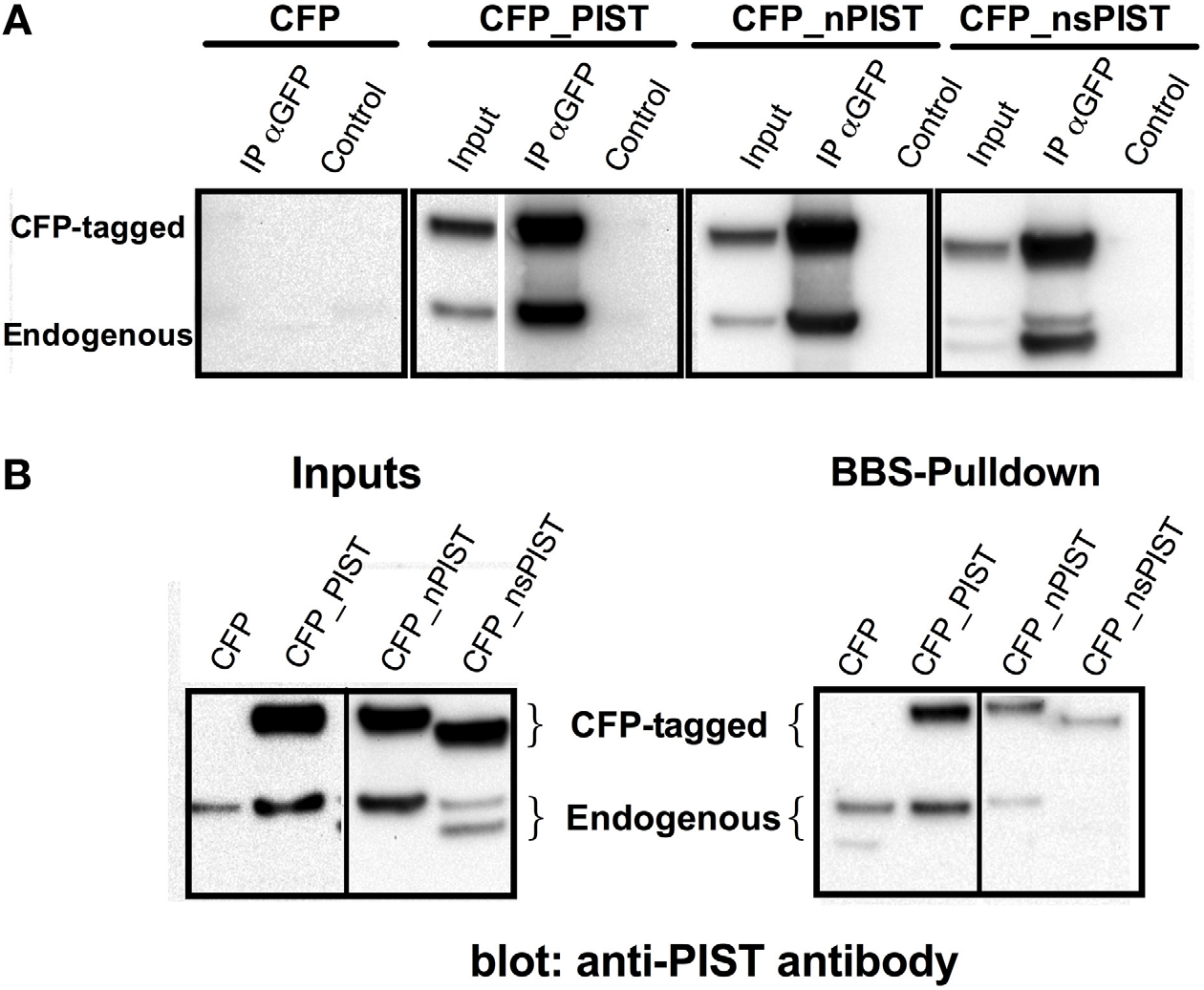
**(A)** Heteromerization of long and short forms of PIST. **(A)** GFP antibody was used to precipitate CFP-tagged PIST isoforms out of lysates of transfected HEK293 cells. PIST isoforms were detected using a polyclonal antibody against PIST. Bands compatible with both the GFP-tagged forms and with endogenous PIST were detected, indicating association between the transfected CFP-PIST and the endogenous one. **(B)** Co-precipitation of PIST isoforms with K_V_10.1_BBS. Lysates of KV10.1_BBS-expressing cells were labeled with an α-BTX-biotin conjugate and pulled down with streptavidin-coated beads (see Figure 1). Anti-PIST specific antibodies detected co-immunoprecipitation of bands compatible with the corresponding CFP-tagged form, together with an endogenous PIST band.

PIST is known to form homodimers depending on the second coiled-coil motif, which bears also a leucine zipper (Neudauer et al., 2001). As this region is intact in every identified isoform, we tested if the shorter variants can form heterodimers with the longer ones. To this end, we transfected CFP-labeled PIST constructs and used a GFP-specific antibody to immunoprecipitate the extracts that were subsequently blotted using a PIST-antibody. Precipitation of CFP-tagged PIST and nPIST co-precipitated a band compatible with endogenous PIST (or nPIST), strongly suggesting that dimerization of PIST occurs in our system (Figure 3A). The major band that co-immunoprecipitated with CFP-tagged nsPIST was smaller than the one pulled by full-length forms and compatible with the size of an endogenous short isoform, suggesting that the short isoforms (or at least nsPIST) do occur at the protein level *in vivo*. Expression of sPIST-CFP was not clearly detectable even after immunoprecipitation, rendering interpretation of the results difficult with this isoform, even though an endogenous band was detected. We therefore excluded sPIST from transfection-based experiments and concentrated on the other three variants.

We cannot exclude formation of heterodimers between nPIST and PIST isoforms, because HEK293 cells express all four isoforms of PIST and in this approach we cannot resolve the eight residues difference between PISTs and nPISTs.

We next tested the different isoforms for their capability to bind K_V_10.1. Precipitation of K_V_10.1_BBS out of stably expressing HEK293 cells was able to co-precipitate over-expressed CFP-tagged PIST and nPIST (Figure 3B). In comparison to the long forms, co-precipitation of the tagged short isoform CFP_nsPIST was much less evident in this assay (Figure 3B). Since the short forms lack most of the PDZ domain, these data suggest an involvement of the PDZ domain of PIST in K_V_10.1 binding. Interestingly, overexpression of the long isoforms resulted in higher intensity in bands compatible with the endogenous full-length PIST, while the shorter isoform rendered more intense bands corresponding to short isoforms (Figure 3B).

### PIST influences currents mediated by K_V_10.1, but not by K_V_10.2

**Figure 4:**
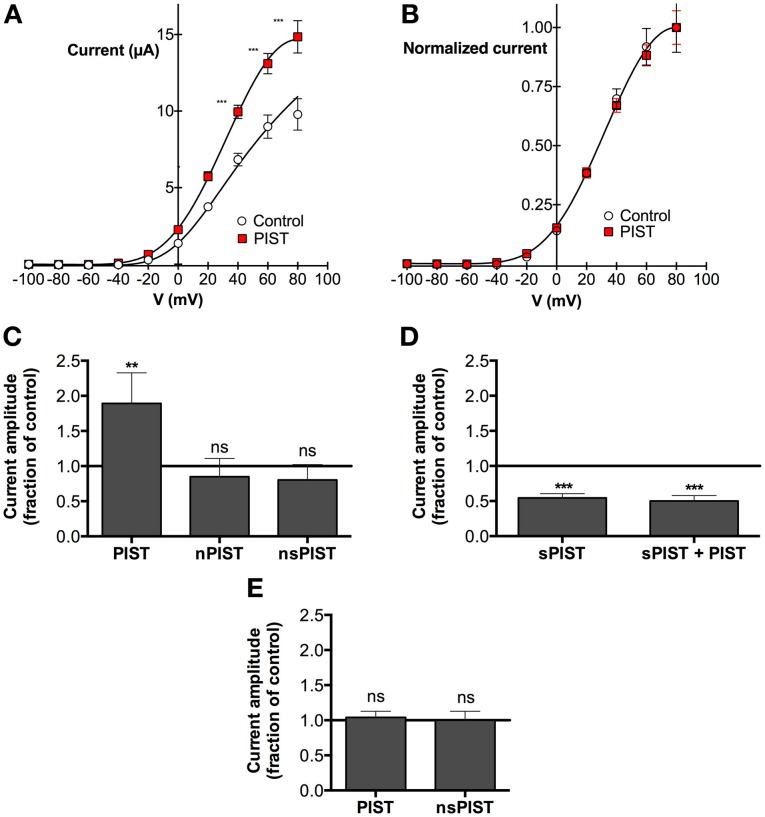
**PIST influences K_V_10.1 function. (A)** Current-voltage relationship of K_V_10.1 expressed in *Xenopus* oocytes with or without PIST. Current amplitudes were significantly larger in oocytes coinjected with PIST (N = 4; ^**^p < 0.01; ^***^p < 0.001). **(B)** After normalization to the current value at +100 mV, the current-voltage plots overlap, indicating that the voltage-dependence of the channel is not altered by PIST. **(C)** Relative current amplitude (at + 80 mV) in oocytes co-expressing PIST isoforms with respect to K_V_10.1 alone (*N* = 19). PIST (*N* = 18) induced a significant increase (*p* < 0.05), while nPIST (*N* = 10) and nsPIST (*n* = 14) did not alter current amplitude. **(D)** Co-injection of sPIST reduced the current amplitude both in the absence (*N* = 8) and in the presence (*N* = 8) of PIST. **(E)** PIST expression did not affect K_V_10.2 current amplitude.

We next tested if there are functional consequences of the interaction between PIST and K_V_10.1. As a model we used voltage clamp recordings in the *Xenopus* oocyte expression system and checked if the overexpression of any PIST isoform can influence K_V_10.1-mediated current. Regardless of the concomitant overexpression of human PIST isoforms, macroscopic K_V_10.1 currents showed very similar voltage dependence (Figure 4); in fact, the current-voltage relationship could be fitted using the same parameters used for K_V_10.1 alone (Figure 4B). We did not appreciate differences in kinetics, and the main properties of K_V_10.1 were conserved in the presence of PIST isoforms (not shown).

The total current amplitude, however, was influenced by PIST expression (Figures 4A,C). Over-expression of PIST was able to nearly double the amplitude of K_V_10.1 mediated current (189 ± 33% of control levels). The difference was more significant the more depolarized the stimulus (reaching *p* < 0.0001 at +80 mV and above). Injection of nPIST and nsPIST did not affect the amplitude. These observations are compatible with an influence of PIST on the membrane expression of K_V_10.1, similar to the one described for CFTR (Cheng et al., 2005). In the presence of PDZ and coiled-coil domain, the amplitude of K_V_10.1 currents was increased. We observed no functional interaction with the insertion in the coiled-coil of “n” forms.

We previously stated that very poor and irregular expression of sPIST did not allow us to document physical interaction between this isoform and K_V_10.1. However, since experiments in oocytes do not depend on transfection or transcription, which is performed *in vitro* previous to oocyte injection, we also tested if injection of synthetic cRNA encoding for sPIST affects the amplitude of the K_V_10.1-mediated current. Surprisingly, sPIST coinjection induced a reduction in current amplitude to 55 ± 4% of control levels. Although they had opposite effects, coinjection of a mixture of PIST and sPIST together with K_V_10.1, did not results on compensation of the effects, but rather sPIST showed a dominant-negative effect, in such a way that the current was reduced to the same extent as when sPIST was injected alone (Figure 4D).

A Golgi-resident protein like PIST might have a general effect on the expression level of ion channels in the oocyte expression system, enhancing transport mechanism toward the membrane. As a measure for the specificity of our observations, we tested the effect of PIST overexpression on K_V_10.2, the closest relative of K_V_10.1. PIST overexpression did not change voltage-dependence or kinetic parameters of K_V_10.2. In contrast to K_V_10.1, there was also no change in current amplitudes of K_V_10.2-mediated currents (Figure 4E), pointing toward a highly specific effect of PIST expression on K_V_10.1, but not K_V_10.2 current amplitude.

### Cellular distribution of K_V_10.1 changes in response to PIST overexpression

**Figure 5:**
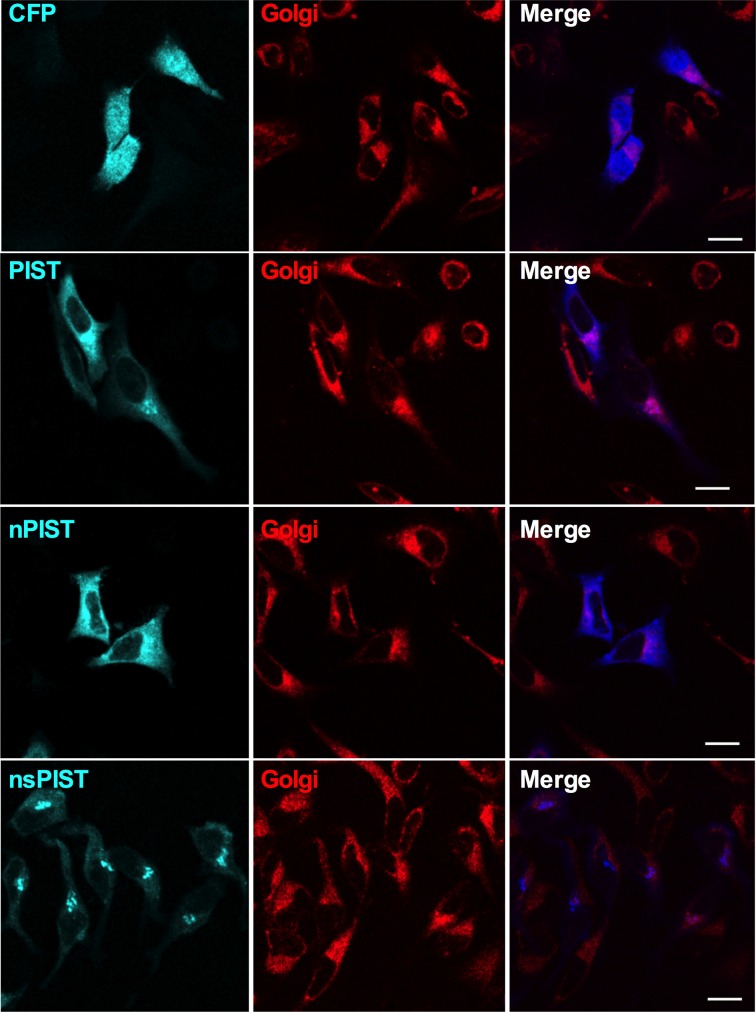
**Single confocal slices of cells expressing CFP-tagged PIST isoforms (blue).** The Golgi apparatus was stained with BODIPY-C5 ceramide (red). Scale bar, 10 μm.

**Figure 6:**
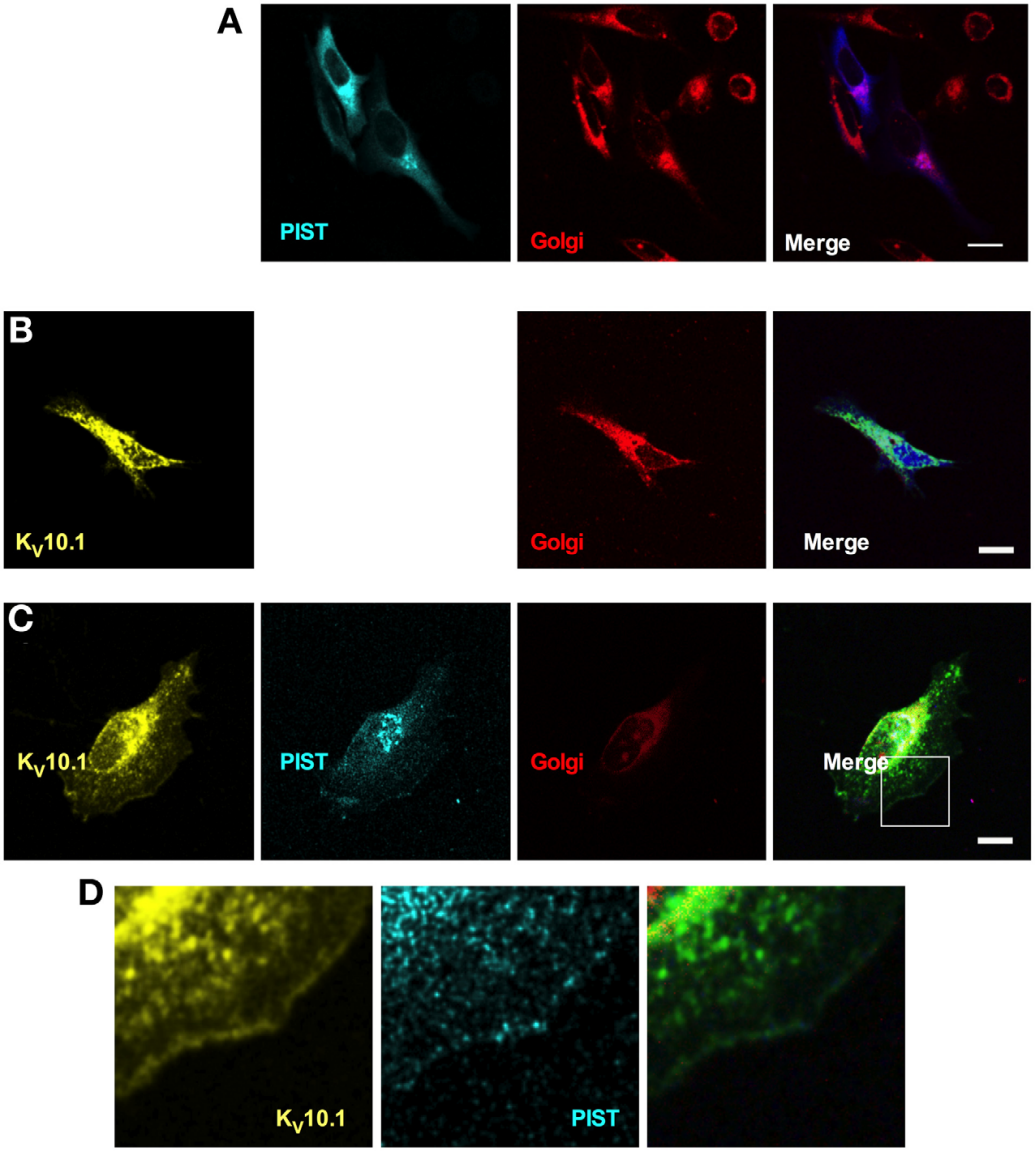
**Cellular distribution of K_V_10.1_Venus and CFP-tagged PIST.** CFP-PIST, when expressed alone **(A)**, reproduced from Figure 5, gave a diffuse distribution with preferential location to the Golgi (stained in red). Overexpressed K_V_10.1 showed a preferentially intracellular distribution **(B)**. When co-expressed **(C)**, both CFP-PIST and K_V_10.1 were detectable at the cell periphery, compatible with an increase of membrane localization of both proteins. **(D)** represents a larger magnification of the area represented by a white square in the “merged” panel in **(C)**, Scale bar: 10 μm.

Having shown that PIST is able to interact physically and functionally with K_V_10.1, we next asked if both proteins co-localize *in vivo*. PIST is reported to reside mainly in the Golgi apparatus, depending on at least one coiled-coil region, but can also be pulled to the membrane in co-expression with its interaction partners (Charest et al., 2001; Yao et al., 2001; Wente et al., 2005b). To perform subcellular localization studies, we used CFP-tagged forms of PIST. As described in the literature, the longer forms of PIST were mainly located in the Golgi, the neuronal form being slightly more diffuse (Figures 5, 6A). nsPIST localized to discrete areas of the Golgi.

Co-expression of a Venus-tagged K_V_10.1 led to a slight change in the localization of PIST only, a fraction of which showed expression at the membrane; this also resulted in a clearer membrane localization of K_V_10.1 itself. Expressed alone, K_V_10.1_Venus showed a typical distribution of this ion channel in the cell, where most K_V_10.1 stays intracellular and only weak expression can be seen at the membrane (Figure 6B). PIST coexpression led to peripheral venus signal compatible with increased channel localization at the plasma membrane (Figures 6C,D). Overexpression of nPIST as well as its short form nsPIST did not change K_V_10.1 localization in the cell (not shown). This data further supports the idea that a trafficking effect of PIST might be involved in the regulation of macroscopic current amplitude.

### PIST changes membrane expression of K_V_10.1

**Figure 7:**
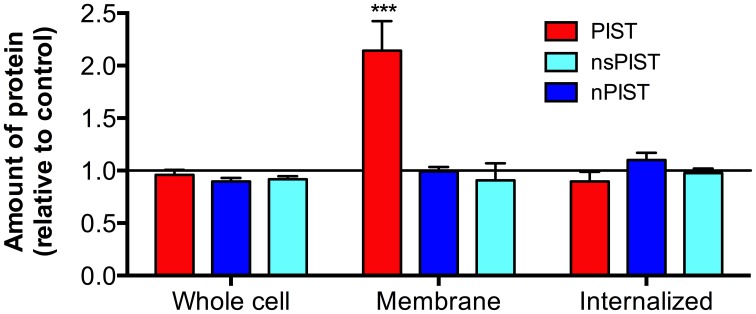
**Over-expression of PIST isoforms differently influences cellular fractions of K_V_10.1_BBS.** Different labeling protocols allowed distinguishing between whole cell, membrane bound and internalized K_V_10.1_BBS by ELISA (see Materials and Methods). The fraction of membrane bound K_V_10.1_BBS, labeled on living cells, was clearly enriched by expression of PIST but not of the other isoforms (*N* = 3; ^***^p < 0.001).

To get more quantitative data we made use of another system recently established in our lab, which is based on K_V_10.1 bearing an α-bungarotoxin (BTX) binding site between transmembrane segments 3 and 4 (K_V_10.1_BBS). Different labeling techniques with an α-BTX-biotin conjugate allow to distinguish between whole cell, membrane bound and internalized K_V_10.1 out of stably expressing cells and to quantify this different fractions via ELISA (Kohl et al., 2011). Specific labeling of membrane K_V_10.1_BBS is possible by incubating intact cells stably expressing K_V_10.1_BBS at low temperature (to avoid internalization) with an α-BTX-biotin conjugate. Quantification of this fraction of membrane bound K_V_10.1_BBS revealed that the overexpression of CFP-tagged PIST can indeed increase surface K_V_10.1_BBS to 214 ± 28% of control levels (Figure 7). In agreement with findings in the oocyte expression system, the neuronal forms of PIST (nPIST and nsPIST) did not have any effect on membrane K_V_10.1_BBS, as values were not different (94 ± 15% nPIST, 91 ± 16% nsPIST) from control levels.

Both increased expression levels and increased stability of plasma membrane K_V_10.1_BBS could explain this observation. To distinguish between both possibilities, we examined the effect of overexpressing PIST isoforms on whole cell K_V_10.1_BBS, labeled after lysis of the cells. Neither isoform of PIST had an effect on the amount of total K_V_10.1_BBS (Figure 7), giving values of 96 ± 5% (PIST), 92 ± 3% (nsPIST) or 102 ± 9% (nPIST) in comparison to control levels, indicating that neither isoform of PIST affect total expression levels of K_V_10.1_BBS. Therefore, they may be involved in the transport of this voltage gated ion channel to and from the plasma membrane.

The K_V_10.1_BBS system easily allows for the quantification of internalized K_V_10.1_BBS within a given time frame (Kohl et al., 2011). For this, we incubated living cells with α-BTX-biotin conjugate at 37°C and allowed internalization for one hour. Removal of the label of membrane-bound K_V_10.1_BBS by acid wash afterwards leaves only the internalized labeled K_V_10.1_BBS for quantification. Interestingly, neither isoform had an effect on the total amount of internalized K_V_10.1_BBS (PIST 90 ± 9%, nsPIST 92 ± 13%, nPIST 98 ± 8%). As membrane expression of K_V_10.1_BBS is clearly increased in response to PIST overexpression (Figure 7), one would expect more channels internalized if the rate of internalization is not affected by this protein.

## Discussion

In this study we identified PIST as a binding partner of K_V_10.1 and cloned two new isoforms of PIST. The novel variants lack a significant fraction of the PDZ domain and are expressed in all tissues examined, although with lower abundance than their respective longer forms. The shorter form was also detected at the protein level in HEK293 cells (Figure 3). The new isoforms correspond to skipping of exon 6 in the human gene, encoding for most of the PDZ domain. Interaction of K_V_10.1 and PIST appears to require the PDZ domain of the latter, since deletion of this domain reduced binding in pull-down assays. On the channel side, the C-terminus of the protein was the part that showed interaction with PIST in a yeast two-hybrid screen, and therefore is a probable mediator of the interaction. However, the interaction might need an additional binding partners, because pull down experiments using recombinant purified proteins (K_V_10.1 C-terminus and PIST) were unsuccessful (not shown).

We found K_V_10.1-mediated current to be influenced by the overexpression of PIST in *Xenopus* oocytes. This effect is apparently not due to changes in the electrophysiological properties of K_V_10.1 but rather due to a change in the surface expression of the channel, which was clearly enhanced by PIST as indicated by surface-labeling experiments. Binding of endogenous PIST was enhanced by overexpression of CFP-tagged PIST, leading to the idea that binding gives a positive feedback that stabilizes or improves association of both proteins *in vivo*.

Forced expression of the short isoform of PIST (sPIST) acts as a dominant negative in *Xenopus* oocytes, in the sense that leads to reduction of functional expression of K_V_10.1 (Figure 4D). These data are in good agreement with previous observations regarding the interaction of PIST with AMPA receptors (Cuadra et al., 2004). Although binding of several proteins to the coiled-coil region of PIST is reported, functional consequences have only been described through interaction with the PDZ domain (Cuadra et al., 2004; He et al., 2004; Cheng et al., 2005; Wente et al., 2005a; Xu et al., 2010). Deletion of the PDZ domain results in a dominant negative effect in AMPA receptor clustering (Cuadra et al., 2004). This led to the hypothesis that the short forms of PIST might serve as negative regulators of PIST function, in good agreement with our observations in oocytes.

nPIST, which differs from PIST by an insertion of eight amino acids, was not able to regulate K_V_10.1 in our model systems. Since the insertion occurs in the coiled-coil region, the lack of regulation by nPIST indicates that, although the physical interaction with K_V_10.1 depends on the PDZ domain, the functional effect requires additionally the coiled-coil region of PIST. This raises the question if and how the surface expression of K_V_10.1, which is normally expressed exclusively in the central nervous system (Pardo et al., 1999), is controlled by nPIST and PIST *in vivo*. We cannot exclude from our data that nPIST might also control surface levels of K_V_10.1 *in vivo* because, as already mentioned, it is likely that additional partners are required the functional interaction of nPIST and K_V_10.1. These additional factor(s) could be different for nPIST and PIST, and its (their) absence might preclude an nPIST-mediated effect in our systems. Our results point to an at least much more efficient influence of PIST compared to nPIST in positively affecting K_V_10.1 surface expression. This implies that, in tumors outside the brain, the surface expression of ectopically expressed K_V_10.1 would be additionally boosted by the expression of PIST.

From our data, we can only speculate about the mechanism by which PIST can influence the surface expression of K_V_10.1. However, we can exclude mechanisms involving an overall increased amount of K_V_10.1, induced either by enhanced synthesis or decreased degradation of the ion channel, since we observed no changes in total K_V_10.1 in cells overexpressing PIST.

Although there are apparently more active channels at the membrane, the amount of internalized K_V_10.1 is not altered by overexpression of PIST, indicating that PIST induces a more efficient transport of K_V_10.1 to the plasma membrane. However, this interpretation might be an oversimplification, because it would require that the amount of K_V_10.1 channels at the plasma membrane does not influence the rate of internalization, which is counterintuitive. Trafficking of K_V_10.1 is not yet completely understood. We have recently described Rabaptin 5 (RABEP1) as an interaction partner of K_V_10.1, linking this ion channel to early endosomes (Ninkovic et al., 2012). As an early event in the maturation and function of early endosomes Rabaptin 5 is able to bind and stabilize the GTP form Rab5, which then recruits effector proteins (Stenmark et al., 1995; Lippe et al., 2001). PIST interacts with Syntaxin 6, a member of the Syntaxin family of SNAREs that is also a Rab5 effector (Charest et al., 2001). Syntaxin 6, together with Syntaxin 13, VTI1a (Vps10p tail interactor 1) and VAMP4 were identified as the SNARE machinery involved in homotypic fusion of early endosomes to where it is recruited by direct binding of early endosomal antigen-1 (Simonsen et al., 1999; Brandhorst et al., 2006; Zwilling et al., 2007). PIST could therefore interfere with this machinery to block K_V_10.1 internalization. Inhibition of internalization processes by overexpression of a dominant-negative point mutant of Rab5(S34N), shown to block endocytosis (Li and Stahl, 1993), drastically reduced the amount of internalized K_V_10.1, but was not able to increase its surface expression significantly (data not shown). If PIST only impairs endocytosis of K_V_10.1, we therefore would not expect such a high increase in its surface expression as seen here.

PIST is also involved in sorting mechanisms, facilitating the trafficking of CFTR to lysosomes when over-expressed (Cheng et al., 2002, 2004, 2005; Gentzsch et al., 2003). Moreover, the Rho GTPase TC10 can bind to PIST and modulate this effect (Neudauer et al., 2001; Cheng et al., 2005). Another example of PIST function is the enhancement of synaptic clustering of AMPA receptors in hippocampal neurons in response to overexpression of the neuronal form of PIST (Cuadra et al., 2004). Deletion of the PDZ domain served as dominant negative form of nPIST clearly reducing AMPA clustering at the membrane (Cuadra et al., 2004). Endosomal sorting of AMPA receptors beginning in early endosomes towards either recycling or late endosomes is well documented to be a main process in long term synaptic plasticity in hippocampal neurons [reviewed in Hanley (2010)]. Therefore nPIST, like PIST for CFTR, might be involved in sorting processes for AMPA receptors, favoring recycling.

A similar effect might be postulated for the K_V_10.1-PIST interaction. PIST may serve as a molecular switch to control the fate of the channel after internalization in early endosomes by enhancing its recycling. This would lead to an increased membrane expression of the channel, while the amount of internalized K_V_10.1 would be reduced, leading in our case to equal amounts of internalized K_V_10.1 despite drastic changes in the membrane expression. Another interaction partner of PIST might regulate this process by binding to the coiled-coil region of PIST, like TC10 does for CFTR (Cheng et al., 2005). Candidate proteins for this interaction are small Rho GTPases like TC10 or Rab proteins, as Rab6a was shown to bind PIST (Bergbrede et al., 2009). The insertion of eight amino acids in the neuronal form of PIST lies in the region putatively responsible for this interaction, leading to the hypothesis that the isoforms can be regulated by different binding partners. These binding partners might decide if a protein bound to the PDZ domain of PIST is sorted to lysosomes or to a recycling process. The lack of the right binding partner for nPIST in our systems would explain why we observed a different behavior of PIST and nPIST although both are able to bind K_V_10.1 in pull down assays. The shorter forms of PIST may serve as a dominant negative variant, whose expression controls PIST function *in vivo* by trapping the binding partners of the coiled-coil region without linking them to effected proteins like K_V_10.1.

### Conflict of interest statement

Luis A. Pardo is shareholder at iOnGen AG. The other authors declare that the research was conducted in the absence of any commercial or financial relationships that could be construed as a potential conflict of interest.
